# 622 Oral Ketamine Administration During Bromelain Based Enzymatic Debridement of Burn Injury

**DOI:** 10.1093/jbcr/irac012.250

**Published:** 2022-03-23

**Authors:** Elizabeth Halicki, Steven A Kahn, Melanie S Condeni, Jason Hirsch, Jonathan Turner

**Affiliations:** Medical University of South Carolina, Hanahan, South Carolina; Medical University of South Carolina, Charleston, South Carolina; Medical University of South Carolina, Charleston, South Carolina; Medical University of South Carolina, Charleston, South Carolina; Medical University of South Carolina, Charleston, South Carolina

## Abstract

**Introduction:**

Existing literature supports bromelain enzymatic debridement as an early tool for selective escharectomy, resulting in fewer skin grafts after burn injury. Enzyme application is painful, so many centers use continuous infusions of narcotics, ketamine, and sedatives in the intensive care unit. The authors utilize oral ketamine in burn practice- as it is safe, can decrease the need for higher level of care, and be used without fasting. The purpose of this study is to evaluate whether oral ketamine can safely be utilized as an alternative to continuous infusions of medications for bromelain treatment.

**Methods:**

This was a retrospective study of patients who received oral ketamine (for analgesia) and oral lorazepam (for anxiolysis and prevention of dysphoria) for bromelain-based selective enzymatic debridement application over a 6-month period. Data collected included patient demographics, burn characteristics, sedatives and analgesics administered, pain scores, adjunct medications, and vital signs. Presence of respiratory insufficiency (desaturations < 93%, need for bag-valve mask, or intubation), hypertensive urgency (SBP >180, DBP >110 w/out end organ dysfunction), dysphoric reactions, and uncontrolled pain were recorded. Oral ketamine (2-4 mg/kg) was given 30 minutes prior to the procedure. Patients were monitored per sedation guidelines and had capnography plus supplemental oxygen. Small PRN IV boluses of narcotics were given during the procedure, along with their baseline oral multimodals and narcotics. Pain was measured with the 1-10 verbal rating scale with minimal pain defined as < 4 or the patient clinically observed as sleeping. Patients were not made NPO for the procedure.

**Results:**

Ten patients were included. Four patients (40%) had minimal pain during enzyme application, 3 (30%) patients slept through their procedure, and 3 (30%) patients reported 10/10 pain during treatment (2 had 10/10 pain before treatment). Five patients were treated on the floor, 7 were treated in the ICU. The median ketamine dose was 225 mg (IQR:177,250), or 3mg/kg (IQR:2.75,3). Additional oral and IV opioids received during the 8–14-hour interval was 21 morphine milligram equivalents (MEs). The median benzodiazepine dosing before and during enzymatic debridement was 1.4 lorazepam MEs. Three patients (30%) had hypertensive urgency, 2 (20%) of whom reported 10/10 pain, and all 3 received 10mg IV labetalol. No one had dysphoric reactions or respiratory insufficiency.

**Conclusions:**

This preliminary study reveals oral ketamine administration is safe and effective for pain control during bromelain-based enzymatic debridement. Most patients required small to moderate amounts of PRN IV opioids, had acceptable pain control, and there were no significant adverse effects. Future large, prospective studies should evaluate dosing and timing for optimal patient outcomes.

